# An *Arabidopsis* Zinc Finger Protein Increases Abiotic Stress Tolerance by Regulating Sodium and Potassium Homeostasis, Reactive Oxygen Species Scavenging and Osmotic Potential

**DOI:** 10.3389/fpls.2016.01272

**Published:** 2016-08-24

**Authors:** Dandan Zang, Hongyan Li, Hongyun Xu, Wenhui Zhang, Yiming Zhang, Xinxin Shi, Yucheng Wang

**Affiliations:** ^1^State Key Laboratory of Forest Genetics and Tree Breeding, Northeast Forestry University, HarbinChina; ^2^Key Laboratory of Biogeography and Bioresource in Arid Land, Xinjiang Institute of Ecology and Geography, Chinese Academy of Sciences, XinjiangChina

**Keywords:** *Arabidopsis thaliana*, AtRZFP, abiotic stress, zinc finger proteins, ROS scavenging analysis, Na^+^ and K^+^ content

## Abstract

Plant zinc finger proteins (ZFPs) comprise a large protein family and they are mainly involved in abiotic stress tolerance. Although *Arabidopsis* RING/FYVE/PHD ZFP At5g62460 (AtRZFP) is found to bind to zinc, whether it is involved in abiotic stress tolerance is still unknown. In the present study, we characterized the roles of AtRZFP in response to abiotic stresses. The expression of AtRZFP was induced significantly by salt and osmotic stress. AtRZFP positively mediates tolerance to salt and osmotic stress. Additionally, compared with wild-type *Arabidopsis* plants, plants overexpressing *AtRZFP* showed reduced reactive oxygen species (ROSs) accumulation, enhanced superoxide dismutase and peroxidase activity, increased soluble sugars and proline contents, reduced K^+^ loss, decreased Na^+^ accumulation, stomatal aperture and the water loss rate. Conversely, *AtRZFP* knockout plants displayed the opposite physiological changes when exposed to salt or osmotic stress conditions. These data suggested that AtRZFP enhances salt and osmotic tolerance through a series of physiological processes, including enhanced ROSs scavenging, maintaining Na^+^ and K^+^ homeostasis, controlling the stomatal aperture to reduce the water loss rate, and accumulating soluble sugars and proline to adjust the osmotic potential.

## Introduction

Zinc-finger proteins (ZFPs) are known to play various important roles in diverse organisms (Luo et al., 2012a), and their expression responds to various abiotic stresses (Sun et al., 2010). According to the number and order of the C and H residues in the secondary structure of the finger, ZFPs are classified into nine groups: C2H2, C8, C6, C3HC4, C2HC, C2HC5, C4, C4HC3, and C3H (Schumann et al., 2007; Gao et al., 2012; Gupta et al., 2012). Based on their structural diversities, ZFPs are categorized into 23 subfamilies, such as GATA, FYVE, TDDP, RBPO, A20, TFIIB, Dof, CDGSH, PADPP, LYAR, ZK, TAZ, MSRING, Ubox, Bbox, NFX1, AN, UBR, Ring, WRKY, DHHC, PHD, and CHY (Li W.T. et al., 2014).

Zinc finger proteins are a large family in plant kingdoms; for instance, there are 176 ZFPs in *Arabidopsis thaliana* and 126 ZFPs in wheat. Although ZFPs are abundant in plants, only a handful of them have been characterized functionally. These characterized ZFPs are involved in plant development (Li X.J. et al., 2014; Seok et al., 2016), regulation of plant height (Liu et al., 2011; Sendon et al., 2014), root development (Liu et al., 2013), flower development (Yang et al., 2014), seed germination (Baek et al., 2015), secondary wall thickening and anther development (Chai et al., 2015), and fruit ripening (Weng et al., 2015). ZFP family proteins are involved in resistance to biotic stresses, such as rice blast fungus infection (Li Y. et al., 2014; Cao et al., 2016). In addition, ZFPs play important roles in abiotic stress. Cheuk and Houde (2016) investigated 53 Q-type C2H2 zinc finger protein (TaZFPs) from *Triticum aestivum*, and showed that these TaZFPs are mainly responsive to high light (44/53), H_2_O_2_ (37/53), drought (37/53), and flooding (31/53); 16 genes were responsive to all stresses tested. This result indicated that these TaZFPs play important roles in abiotic stress resistance. A study of 109 C2H2 zinc-finger (C2H2-ZF) proteins from *Populus trichocarpa* showed that most of them contain phytohormone or abiotic stress-related *cis*-elements in their promoter regions. Quantitative real-time RT-PCR analysis suggested that these C2H2-ZF genes are involved significantly in salt, drought, and heat responses (Liu et al., 2015). Abiotic stress alters the metabolic balance to induce the generation of reactive oxygen species (ROS; Miller et al., 2008). Excess ROS causes oxidative damage to macromolecules, including DNA, proteins and lipids. Damage to macromolecules will lead to modulation of gene expression, the cell cycle, cell metabolism, cell adhesion, and cell death, ultimately interfering with their normal function (Kadir et al., 2013). Therefore, control of the ROS level is critical for abiotic tolerance. ZFPs plays important roles in ROS scavenging resulting from abiotic stress. ZFPs regulate the expression of a series of stress-activated genes in plants via ROS signaling to resist salt, drought or oxidative stress. Genes involved in antioxidation are regulated significantly by ZFPs, which leads to elevated antioxidant enzyme activities and reduced ROS accumulation; thus, enhancing abiotic stress tolerance (Davletova et al., 2005; Sun et al., 2010; Zhang et al., 2014; Baek et al., 2015; Fan et al., 2015). In addition to ROS pathways, other physiological adaptations to abiotic stress tolerance mediated by ZFPs have been studied. ZFPs can confer abiotic stress tolerance by increasing the contents of abscisic acid (ABA), proline, soluble sugars or chlorophyll, and reducing the water loss rate (Luo et al., 2012b; Wang et al., 2016). ZFPs are also involved in gibberellins (GA) signaling in the regulation of the stress response. Transgenic plants overexpressing the ZFPs OsDOG (Liu et al., 2011) and ZFP185 (Zhang Y. et al., 2016) displayed a reduced GA content, dwarf phenotypes and sensitivity to abiotic stress. However, plants overexpressing BBX24 (Yang et al., 2014) also showed reduced GA content, but plants expressing BBX24 showed normal growth phenotype and enhanced salt and drought stress tolerance (Yang et al., 2014). ZFPS are also involved in high temperature (Kim et al., 2015) and H^+^ tolerance (Fan et al., 2015). Although many ZFPs regulate abiotic stress tolerance positively, some ZFPs regulate abiotic stress tolerance negatively. For example, arginine-rich tandem zinc-finger proteins (RR-TZF) AtTZF3 regulate seed germination negatively under salt stress conditions (D’Orso et al., 2015). Soybean GmZFP3 might be involved in the ABA-dependent signaling pathway, and plays a negative role in drought tolerance (Zhang D. et al., 2016). Analysis of ABA-related marker gene expression in *Arabidopsis* suggested that GmZFP3 might be involved in the ABA-dependent pathway during the drought stress response. OsDOG and ZFP185 also play negative roles in abiotic stress tolerance (Liu et al., 2011; Zhang Y. et al., 2016).

Additionally, ZFPs also interact with different proteins to mediate abiotic stress tolerance. For instance, Bogamuwa and Jang (2016) showed that Tandem CCCH Zinc Finger protein (ZFP), AtTZF could interact with the proteins such as Mediator of ABA-Regulated Dormancy 1 (MARD1) and Responsive to Dehydration 21A (RD21A) to perform its functions in ABA, GA, and phytochrome-mediated seed germination responses. Zinc Finger of *A. thaliana*12 (ZAT12) is induced by abiotic stress and interacts with FER-LIKE IRON DEFICIENCY-INDUCED TRANSCRIPTION FACTOR (FIT). Under Fe deficient conditions, H_2_O_2_ levels were increased in a FIT-dependent manner, and the FIT protein was stabilized by H_2_O_2_ in the presence of ZAT12. ZAT12 has a negative role in Fe acquisition mediated by H_2_O_2_ signaling in the Fe deficiency responses (Le et al., 2016). ZAT6 interacts with, and is phosphorylated by, a stress-responsive mitogen-activated protein kinase, MPK6. Phosphorylation of ZAT6 by MPK6 is necessary for ZAT6’s role in seed germination under salt and osmotic stress (Liu et al., 2013).

Previously, to identify the RING/FYVE/PHD ZFP involved in abiotic stress, we acquired some RING/FYVE/PHD ZFPs T-DNA insertion mutants from the *Arabidopsis* Biological Resource Centre (ABRC). We use DAB and Evans blue staining of these mutants to screen them initially for those that were sensitive to salt and mannitol stress. According to DAB and Evans blue staining, At5g62460 mutant plants (SALK_119330) was sensitive to salt stress compared with WT plants, implying that At5g62460 (AtRZFP) might be involved in the abiotic stress response, and was selected for further study. Our study showed that AtRZFP could increase tolerance to salt and osmotic stress, and we further revealed the physiological changes modulated by AtRZFP in response to abiotic stress. Our results provide useful insights into the function of ATRZFP in the regulation of salt and osmotic stress tolerance in *A. thaliana*.

## Materials and Methods

### Plant Materials, Growth Conditions, and Treatments

*A. thaliana* Columbia type (Col) plants were used in this study. The T-DNA insertion At5g62460 mutant (SALK_119330) was obtained from the ABRC. Seeds were surface sterilized and seeded on 1/2MS solid medium containing 2% sucrose at 22°C under a 16 h light/8 h dark photoperiod. To analyze the expression of AtRZFP in response to abiotic stress, 4-week-old *Arabidopsis* plants were watered with a solution of 150 mM NaCl or 200 mM mannitol on their roots. After treatment for 3, 6, 12, and 24 h, the roots and aerial parts of plants were harvested for expression analysis. Plants watered with fresh water were harvested at corresponding time points as controls.

#### Plasmid Constructs and Plant Transformation

The coding sequence (CDS) of ZFP (AtRZFP) was cloned into the pROK2 vector (Hilder et al., 1987) under the control of CaMV 35S promoter to generate the 35S:ZFP construct, and was transformed into *Arabidopsis* plants using the flower-dipping method. Six transgenic lines were obtained (OE lines). The diagram of 35S:AtRZFP construct used for transformation is shown in **Figure 2A**. The AtRZFP knockout lines (SALK_119330) were homozygous for two generations, and the diagram of the T-DNA insertion positions in the SALK_119330 mutant plant is shown in **Figure 2C**, which represent a single allele of At5g62460. The expression of *AtRZFP* in (OE) lines and the SALK_119330 individual plants was monitored using quantitative real-time reverse transcription PCR (qRT-PCR) analysis.

#### Subcellular Location and Western Blotting Analysis

The CDS of AtRZFP, without its stop codon, was ligated in-frame to the N-terminus of the green fluorescent protein (GFP) under the control of CaMV 35S promoter (35S:ZFP-GFP). GFP under control of 35S promoter was also generated (35S:GFP). All primers used to make these constructs are shown in Supporting Information Supplementary Table S1. For subcellular location analysis, the constructs 35S:AtRZFP-GFP and 35S:GFP were introduced separately into onion epidermal cells using particle bombardment (Bio-Rad, Hercules, CA, USA). The transformed cells were analyzed under an LSM700 confocal laser microscope (Zeiss, Jena, Germany). GUS activity analysis was performed (Jefferson et al., 1987). For western blotting analysis, proteins were isolated from onion epidermal cells expressing 35S:AtRZFP-GFP transformed via particle bombardment or non-transformed onion epidermal cells (control). Western blot was performed according to the procedures described by Han et al. (2005), and an anti-GFP antibody (Beyotime, Shanghai, China) was used to detect 35S:AtRZFP-GFP.

### *AtRZFP* Expression Assays in Response to Treatments

*Arabidopsis* seedlings were grown in a greenhouse under normal conditions. *AtRZFP* gene expression in response to various treatments was analyzed. Roots and leaves of 4-weeks-old seedlings were treated with 150 mM NaCl or 200 mM mannitol. Roots and leaves were sampled at 3, 6, 12, and 24 h after each treatment, and well watered plants were harvested at the corresponding time points as controls for RNA extraction.

### Stress Tolerance Assays

T_3_ generation lines of the *AtRZFP* transformed *Arabidopsis* were randomly selected (OE 3 and OE 4) for stress tolerance. For the germination rate assay, the seeds were sown on half-strength Murashige–Skoog (1/2 MS) medium (as control), or 1/2 MS supplied with 150 mM NaCl or 200 mM mannitol. After 1 week, the germination rates of the OE transgenic lines, wild-type (WT) and two SALK_119330 individual plants (KO 4 and KO 5) were calculated. For the stress tolerance assay, seeds were sown on 1/2 MS medium for 3 days. After germination, the seeds were transferred into 1/2 MS medium (as the control condition) or 1/2 MS medium supplied with 150 mM NaCl or 200 mM mannitol. After 2 weeks, the root length and fresh weights (FWs) were measured. The experiments were conducted with three independent biological replications.

### qRT-PCR Analysis

Total RNA was isolated from *Arabidopsis* using the Trizol reagent (Promega, Madison, WI, USA), reversely transcribed into cDNA using the Primescript^TM^ RT reagent kit (Takara), and diluted with ultra-pure water (MilliQ) to 100 μl as the PCR template. The tubulin β-2 (Locus number AT5G62690) and *Actin 7* (Locus number AT5G09810) genes were used as internal references. All primers used are shown in Supplementary Table S2. qRT-PCR was carried out on an Opticon 2 System (Bio-Rad). The PCR reaction mixture contained 10 μl of SYBR Green Real-time PCR Master Mix (Toyobo), 0.5 μM of each forward and reverse primers, and 2 μl of cDNA template (equivalent to 20 ng of total RNA) in a reaction volume of 20 μl. The PCR conditions were as follows: 94°C for 30 s; followed by 45 cycles at 94°C for 12 s, 60°C for 30 s, 72°C for 40 s; and 1 s at 82°C for plate reading. A melting curve of each sample was generated to evaluate the quality of the amplified product. Three biological replicates were conducted, and the expression levels were calculated using the 2^-ΔΔCt^ method.

### Detection of ROS Accumulation and Cell Death using Biochemical Staining

Detached leaves from different *Arabidopsis* lines were treated with 200 mM NaCl or 300 mM mannitol for 0, 6, and 12 h, and then used for histochemical staining analysis. The leaves were infiltrated with DAB or NBT, which allowed the detection of H_2_O_2_ and O_2_^-^, respectively, according to the method (Fryer et al., 2002). Evans Blue staining was performed to investigate the cell death, as described (Zhang L. et al., 2011). H_2_O_2_ was measured following the protocol of Thordal-Christensen et al. (1997).

### Analysis of Physiological Changes Involved in Abiotic Stress Tolerance

For the physiological studies, OE, WT, and two SALK_119330 individual plants were treated with 150 mM NaCl or 200 mM mannitol for 5 days and harvested for analysis. Measurement of the activities of SOD and POD were conducted according to the description (Dong and Chin, 2000). The MDA content was determined (Dhindsa et al., 1981). Electrolyte leakage was measured following the protocol (Sreenivasulu et al., 2000). Proline content was measured (Bates et al., 1973).

To measure the soluble carbohydrate content, 0.5 g of sample was ground into a fine powder, and was added with 5 ml of 80% ethanol at 80°C for 30 min. The extracting solution was then centrifuged at 12000 rpm for 10 min. After centrifugation, the supernatant was diluted with 80% ethanol to 10 mL. One mL of the mixture and 5 mL anthrone were heated in boiling water for 15 min, cooled immediately and placed in the dark for 30 min. The absorbance of the supernatant was detected at 620 nm, with water as the background. One sample per line was quantified three times.

To determine the Na^+^ and K^+^ contents, 1 week-old *Arabidopsis* seedlings were grown in a greenhouse supplemented without or with 150 mM NaCl. The leaves and roots were then dried in an oven at 80°C for 48 h for weighting. The dried sample (0.1 g) was added with 10 mL deionized water, heated in boiling water for 2 h, cooled immediately and centrifuged at 12000 rpm for 15 min. After centrifugation, the supernatant was used for cation content determination and water was used as the background in an atomic absorption spectrophotometer, as described previously (Chen et al., 2005; Ma et al., 2011).

To assay the water loss rate, the detached leaves from each line were weighed immediately as the FW, and incubated on a clean bench at a relative humidity of about 50%, and their weights were measured at designated time intervals as the desiccated weight. After measurement, leaves were oven-dried at 80°C to a constant dry weight (DW). Water loss rates (%) were calculated according to the formula: Water loss rate (%) = 1–(desiccated weight–DW)/(FW–DW) × 100.

Stomatal apertures were measured according to Pei et al. (1997). Epidermal strips from leaves of different lines were floated in a solution containing 0 mM (control) or 150 mM NaCl, 10 mM Mes-KOH, and pH 6.15 for 3 h under light (150 μmol⋅m^-2^⋅s^-1^) at room temperature, after which they were observed under a light microscope (Olympus BX43, Japan). Leaves similar in size were taken from different lines at the same time. Three leaves were selected from each line or plant, 3 different epidermis parts were observed in each leaf, and 10 stomata guard cells in each epidermis part were measured. The length and width of the stomatal apertures were determined using the software Image J^1^. The ratios of width to length were then calculated.

### CoroNa-Green Staining

To visualize the Na^+^ distributions in the root cells of Col-0, OE lines and two SALK_119330 individual plants, the intracellular Na^+^ specific fluorescent indicator CoroNa-Green AM (Invitrogen Corp, Carlsbad, CA, USA) was used. Five-day-old seedlings grown on 1/2 MS medium were transferred to fresh medium containing 0 mM (control) or 150 mM NaCl for 72 h. The seedlings were then washed 2–3 times with distilled water and stained with 10 μM CoroNa-Green AM in the presence of pluronic acid (0.02%, Invitrogen) for 3 h. The incubated sections were visualized under an LSM700 microscope (Zeiss, Jena, Germany) at excitation and emission wavelengths of 488 and 516 nm, respectively, as described by Oh et al. (2010). The experiments were conducted with three independent biological replications.

### Statistical Analyses

Statistical analyses were conducted using the SPSS 16.0 software package (SPSS Inc, Chicago, IL, USA). Data were compared using one-way ANOVA and differences were considered statistically significant d at *P* < 0.05.

## Results

### The Expression of *AtRZFP* Is Induced by NaCl and Mannitol

**FIGURE 1:**
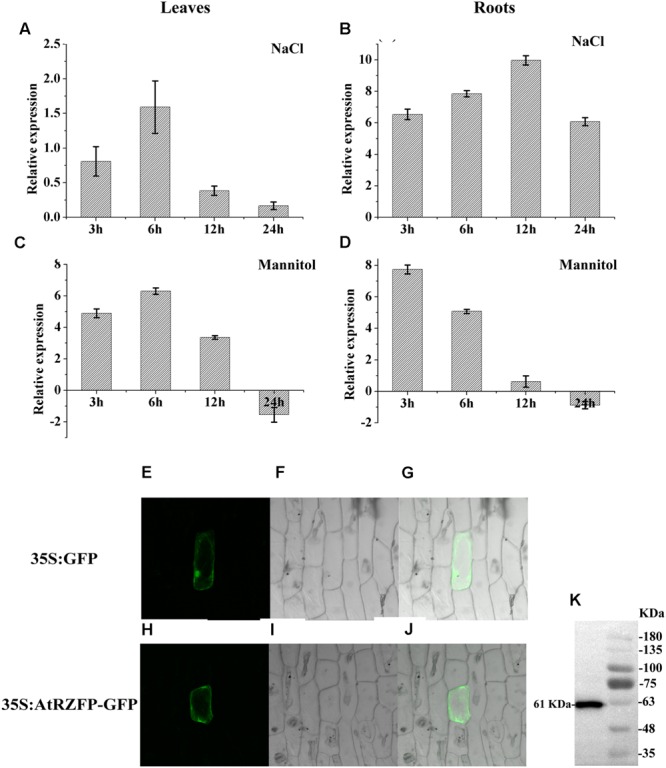
**Expression and subcellular localization of AtRZFP. (A–D)** Well watered plants were harvested at each stress time point as controls. The expression of AtRZFP at each treatment time point was normalized by the expression of the AtRZFP transcript in the control harvested at the corresponding time. The expression data were log2 transformed. **(E–J)** The fusion construct for 35S:AtRZFP-GFP and the 35S:GFP control vector were introduced into onion epidermal cells by particle bombardment. **(E,H)** GFP fluorescence; **(F,I)** onion peel cells imaged under bright field illumination; **(G,J)** the merged images of bright-field and GFP; **(K)** Western blotting analysis of the expression of the AtRZFP-GFP protein. The full-length AtRZFP-GFP protein with the expected size of 61 kDa was detected.

To study the expression of *AtRZFP* in response to NaCl and mannitol stress, real-time RT-PCR was carried out. In leaves, the expression of *AtRZFP* was induced greatly by NaCl or mannitol stress for 3 and 6 h, but decreased as stress continued for 12 and 24 h (**Figures 1A,C**). Interestingly, *AtRZFP* showed similar expression patterns in leaves when exposed to NaCl or mannitol stress (**Figures 1A,C**). The expression of *AtRZFP* was highly induced by NaCl in roots under NaCl stress for 3 to 24 h, and reached a peak at 24 h (**Figure 1B**). In roots, the expression of *AtRZFP* was highly induced when exposed to mannitol stress for 3 h, but decreased thereafter (**Figure 1D**). These results showed that the expression of *AtRZFP* responded to salt and osmotic stress, suggesting that AtRZFP plays a role the abiotic stress response.

### AtRZFP Is Located to Cytoplasm and Membrane

To investigate the subcellular location of AtRZFP, the construct of 35S:AtRZFP-GFP was produced and transformed into onion epidermal cells. The green fluorescent signal from AtRZFP-GFP was observed clearly in the cytoplasm and membrane, while the 35S:GFP control was distributed uniformly throughout the cells (**Figures 1E–J**). Meanwhile, western blotting showed that the AtRZFP-GFP protein had been successfully expressed with no degradation (**Figure 1K**). These results demonstrated that AtRZFP localizes to the cytoplasm and membrane.

### Generation of *AtRZFP* Overexpressing or Knockout Plants

**FIGURE 2:**
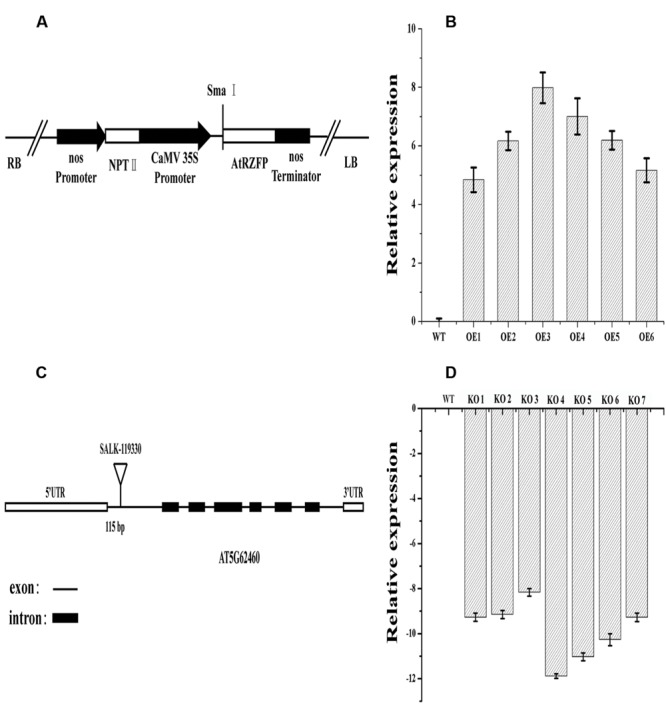
**Analysis of AtRZFP transcript levels in overexpression (OE) lines and Knockout of AtRZFP (KO) plants. (A)** Diagram of the plant expression vector for the overexpression of AtRZFP. **(B)** The expression of AtRZFP in OE lines. **(C)** Diagram of the T-DNA insertion positions of T-DNA insertion positions for a single allele At5g62460. **(D)** The expression of AtRZFP in the KO plants (SALK_119330 individual plants). The expression level of AtRZFP in each line was normalized by the expression of AtRZFP in wild-type (WT) plants. All the expression data were log2 transformed.

The results showed that the expression level of *AtRZFP* was significantly elevated in all OE transgenic lines, and two independent T_3_ homozygous lines (OE 3 and OE 4 lines) with highest (increased by 256-fold) and middle (increased by 128-fold) expression levels, respectively, were selected for subsequent studies (**Figure 2B**). Seven SALK_119330 individual plants (termed KO 1–7) were obtained, and the expression of *AtRZFP* was analyzed in these plants. The results showed that the expression of *AtRZFP* in all these seven individual plants was downregulated, and two individual plants, KO 4 and KO 5, which had the lowest expression levels, were selected for subsequent studies (**Figure 2D**).

### *AtRZFP* Increases Salt and Osmotic Stress Tolerance

**FIGURE 3:**
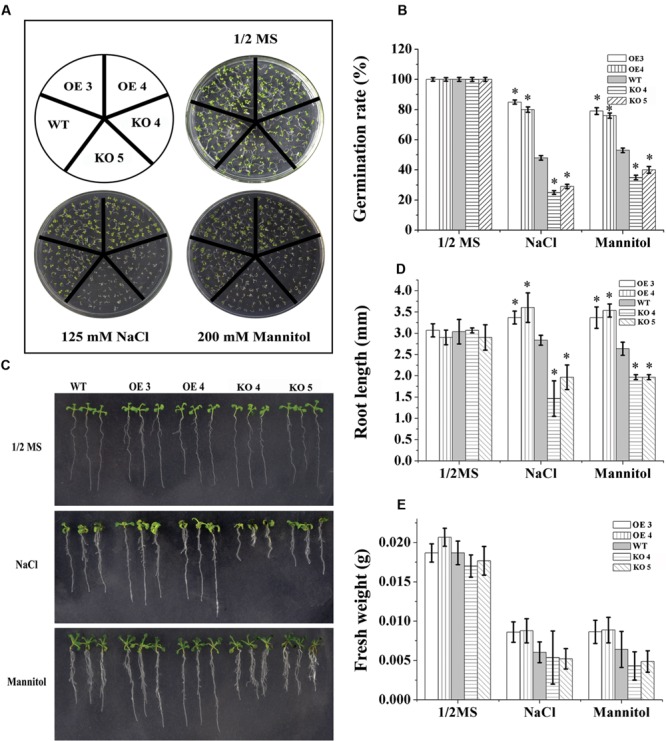
**Tolerance Analysis of AtRZFP. (A,B)** Seed germination rate analysis. Seeds were grown in 1/2 or 1/2 MS supplied with 150 mM NaCl or 200 mM mannitol for 1 week, after which, the germination rates were compared. **(C)** Comparison of growth phenotypes. **(D)** Root length. **(E)** Analysis of fresh weight (FW). Three independent experiments were performed; bars indicate standard deviation. *Indicates significant differences between the OE lines and WT, or between WT and the SALK_119330 individual plants (KO 4 and KO 5) under the same conditions (*P* < 0.05).

To characterize the function of AtRZFP in abiotic stress tolerance, three types of plants with different *AtRZFP* expression levels were studied, i.e., the WT plants, two independent T_3_ homozygous lines overexpressing AtRZFP, OE 3 and 4, and two AtRZFP mutant lines (KO 4 and KO 5). Under normal growth conditions, no difference in seed germination rates, growth phenotype, FWs and root lengths were observed among the studied lines (**Figure 3**), indicating that overexpression or knockout of *AtRZFP* in *Arabidopsis* plants does not affect seed germination, growth rate and phenotype. However, after treatment with NaCl or mannitol, the two OE lines had significantly higher seed germination rates, FWs and root lengths compared with the WT plants; whereas both KO 4 and KO 5 had lower of seed germination rates, FWs and root lengths compared with the WT plants (**Figure 3**). These results indicated that overexpression of *AtRZFP* could increase tolerance to salt and osmotic stress.

### ROS Accumulation and Scavenging Analysis

**FIGURE 4:**
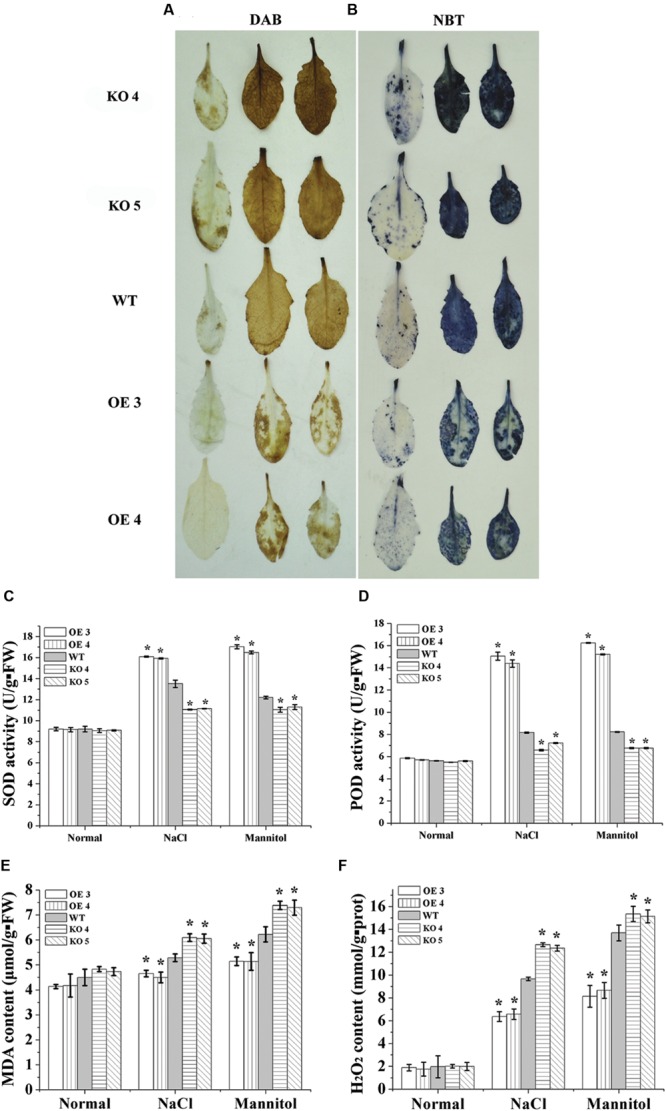
**Reactive oxygen species (ROS) scavenging analysis. (A,B)** Detection of ROS by Diaminobenzidine (DAB) and nitroblue tetrazolium (NBT) staining. Leaves treated with NaCl or mannitol were stained with DAB **(A)** or NBT **(B)** to detect H_2_O_2_ and O_2_^-^, respectively. **(C,D)** Analysis of superoxide dismutase (SOD) **(C)** and peroxidase (POD) activities **(D)**. **(E)** Comparison of malondialdehyde (MDA) contents among OE lines, WT and two SALK_119330 individual plants (KO 4 and KO 5). **(F)** Determination of H_2_O_2_ contents. The experiments were conducted with three independent biological replications. *Indicates significant differences between the OE lines and WT, or between WT and the SALK_119330 individual plants (KO 4 and KO 5) under the same conditions (*P* < 0.05).

DAB and NBT staining were performed to study the accumulation of H_2_O_2_ and O_2_^-^. Compared with the WT plants, both H_2_O_2_ and O_2_^-^ were reduced greatly in the leaves of the two OE lines under salt and osmotic stress conditions (**Figures 4A,B**). Conversely, H_2_O_2_ and O_2_^-^ in the KO 4 and KO 5 individual plants were substantially higher than that in the WT plants (**Figures 4A,B**). We further determined H_2_O_2_ level, and the results showed that H_2_O_2_ content was highest in KO 4 and KO 5 plants, followed by in WT plants, and lowest in the two OE lines, which is consistent with DAB staining (**Figure 4F**). We further studied whether the altered ROS level was caused the changed antioxidant activity in the plants. The levels of two main antioxidant enzymes, SOD and peroxidase (POD), which are involved in ROS scavenging, were determined. Under normal conditions, the activities of SOD and POD were similar among the studied plants (**Figures 4C,D**). Under NaCl and mannitol treatment conditions, compared with the WT plants, both SOD and POD activities were significantly increased in the OE lines, but decreased in the KO 4 and KO 5 plants (**Figures 4C,D**). Correspondingly, the malondialdehyde (MDA) content analysis showed that there was no difference in MDA contents among the studied lines under normal conditions (**Figure 4E**). When exposed to salt or osmotic stress conditions, the MDA content in all the studied plants increased. However, the OE lines displayed lower MDA contents than the WT plants, while the KO 4 and KO 5 had the highest MDA contents among the studied lines (**Figure 4E**). Therefore, AtRZFP could decrease membrane lipid peroxidation generated by salt or osmotic stress to improve salt and osmotic stress.

### Electrolyte Leakage and MDA Content Analysis

**FIGURE 5:**
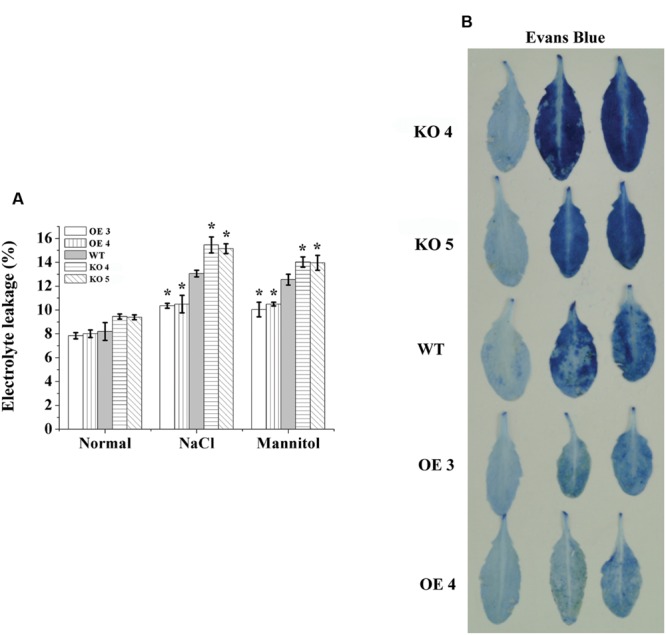
**Electrolyte leakage analysis. (A)** Comparison of electrolyte leakage rates. **(B)** Evans blue staining assay. Three independent experiments were averaged; bars indicate standard deviation. *Indicates significant differences between the OE lines and WT, or between WT and the SALK_119330 individual plants (KO 4 and KO 5) under the same conditions (*P* < 0.05).

Electrolyte leakage was determined to monitor the level of cell death. No significant differences in electrolyte leakage rates were observed among the studied lines under normal conditions. However, under salt and osmotic stress conditions, electrolyte leakage among the OE lines, KO 4, KO 5, and WT plants were significantly different. Electrolyte leakages in the KO 4 and KO 5 individual plants were highest, followed by the WT, and then the OE lines, which had the lowest electrolyte leakage (**Figure 5A**). Consistently, Evans blue staining also confirmed that cell membrane damage was increased greatly in the KO 4 and KO 5 individual plants, but was substantially decreased in the OE lines (**Figure 5B**). These results indicated that overexpression of *AtRZFP* could reduce cell death to improve salt or osmotic stress tolerance.

### Na^+^ and K^+^ Content

**FIGURE 6:**
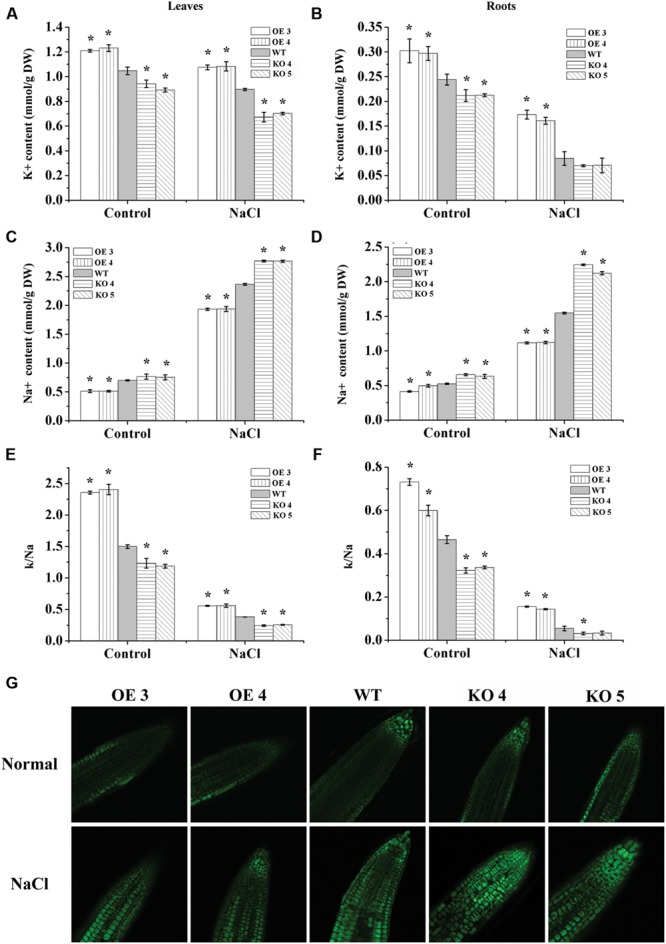
**Analyses of Na^+^ and K^+^ contents.** One week-old *Arabidopsis* seedlings grown in a greenhouse were treated with 150 mM NaCl for 7 days, and plants grown under normal conditions were used as controls. **(A,B)** K^+^ content in leaves **(A)** and roots **(B)**. **(C,D)** Na^+^ content in leaves **(C)** and roots **(D)**. **(E,F)** K^+^/Na^+^ ratio in leaves **(E)** and roots **(F)**; *indicates significant differences between the OE lines and WT, or between WT and the SALK_119330 individual plants (KO 4 and KO 5) under the same conditions (*P* < 0.05). **(G)** CoroNa-Green staining analysis of Na^+^ accumulation under normal or salt stress conditions. Na^+^ was stained by CoroNa-Green and shown as green fluorescence; the root tips of each line were observed. The experiments were conducted with three independent biological replications.

The contents of Na^+^ and K^+^ were determined in the plant leaves and roots. The results showed that under normal conditions, the OE lines also had a relative lower Na^+^ level, higher K^+^ level and higher K^+^/Na^+^ ratio than the WT plants; conversely, the KO 4 and KO 5 individual plants had a higher Na^+^ content, lower K^+^ level and K^+^/Na^+^ ratio than the WT plants (**Figure 6**). When exposed to salt stress conditions, all the studied lines showed reduced K^+^ contents under salt treatments, but both the OE 3 and OE 4 lines still had the highest K^+^ content in their leaves and roots, followed by that in the WT plants, whereas both KO 4 and KO 5 lines had the lowest K^+^ contents. All the studied lines showed increased Na^+^ content under salt stress conditions. However, the KO 4 and KO 5 lines accumulated significantly higher Na^+^ contents than the WT and OE plants, and both OE lines had significant lower Na^+^ accumulation than the WT plants (**Figure 6**). Additionally, both OE lines had the highest K^+^/Na^+^ ratio, followed by the WT, with the two SALK_119330 individual plants (KO 4 and KO 5) having the lowest K^+^/Na^+^ ratio under salt stress conditions.

### Na^+^ Distribution Assay using CoroNa-Green Staining

We further studied the accumulation and distribution of Na^+^ in roots at different lines using CoroNa-Green staining. The results showed that the OE lines had relatively lower Na^+^ contents than the WT and KO 4 and KO 5 plants under normal conditions. When exposed to salt stress, the KO 4 and KO 5 individual plants showed obviously higher Na^+^ accumulation than the WT plants, but the OE lines showed obviously lower Na^+^ accumulation than the WT plants (**Figure 6G**). These results were consistent with the measurement of Na^+^ using the flame photometer (**Figure 6**), indicating that AtRZFP could regulate the accumulation of Na^+^ in plants in response to salt stress.

### Expression of *AtRZFP* Decreased Stomatal Apertures to Reduce Water Loss

**FIGURE 7:**
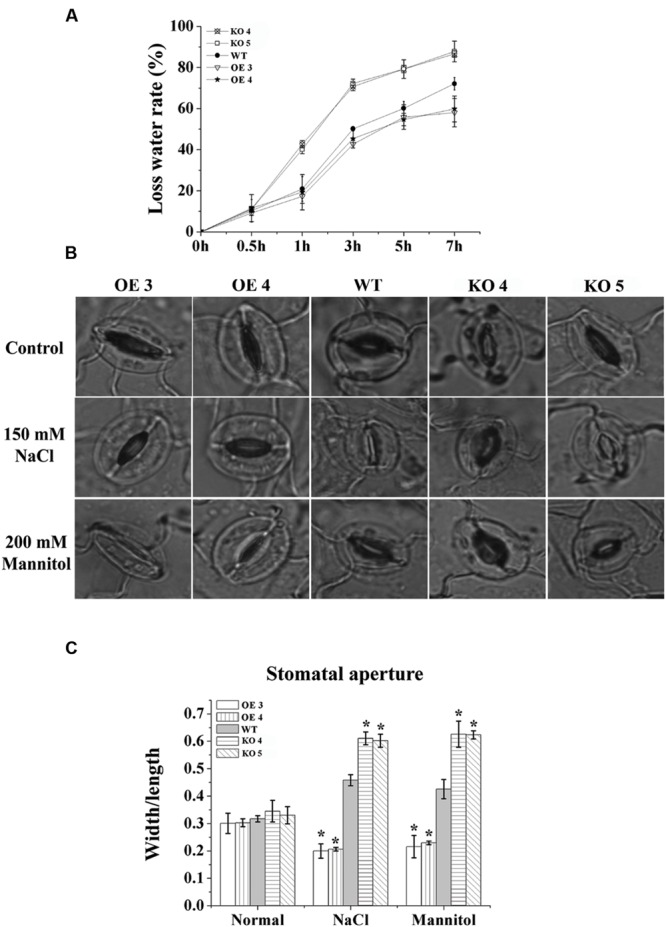
**Analysis of water loss rates and the stomatal aperture. (A)** Water loss rates. **(B)** The stomatal aperture in each line under normal, salt or mannitol stress conditions. **(C)** Calculation of the width/ length ratio of the stomatal aperture in each line. The experiments were conducted with three independent biological replications. *Indicates significant differences between the OE lines and WT, or between WT and the SALK_119330 individual plants (KO 4 and KO 5) under the same conditions (*P* < 0.05).

To study whether the conservation of water capability is altered by the changed expression of AtRZFP, water loss rates were calculated. Compared with the WT plants, the water loss rates of the OE lines were decreased significantly; however, the water loss rates of the KO 4 and KO 5 individual plants were increased greatly (**Figure 7A**).

To investigate whether the reduced water loss rate was caused by changes to stomatal apertures, we measured the stomatal apertures in leaves of the studied lines. Under normal conditions, the stomatal apertures of the studied lines were similar (**Figures 7B,C**). However, under salt or osmotic stress conditions, compared with the WT plants, both OE lines showed the smallest stomatal apertures (width/length), and the KO 4 and KO 5 individual plants showed the highest stomatal apertures (**Figures 7B,C**), indicating that AtRZFP is involved in modulating the closure and opening of stomatal apertures in response to salt and osmotic stress conditions.

### Proline and Soluble Sugar Content Analysis

**FIGURE 8:**
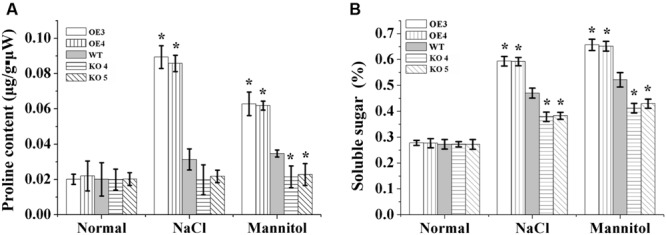
**Analysis of proline biosynthesis and soluble sugar mediated by AtRZFP contents under NaCl and mannitol treatment conditions. (A)** Analysis of the proline level in OE lines, WT and KO 4 and KO 5 plants. **(B)** Analysis of soluble sugar contents in OE lines, WT and KO 4 and KO 5 plants. Three independent biological replications were performed; bars indicate the standard deviation. *Indicates significant differences between the OE lines and WT, or between WT and the SALK_119330 individual plants (KO 4 and KO 5) under the same conditions (*P* < 0.05).

There was no difference in the proline contents among the OE lines, KO 4, KO 5 and WT under normal growth conditions. After salt or mannitol treatments, the WT and OE lines showed increased proline levels, while the proline levels in the KO 4 and KO 5 individual plants did not change. Additionally, in the OE lines, the proline contents increased significantly compared with the WT plants, and the proline contents in the KO 4 and KO 5 plants were lower than in the WT lines under salt and osmotic stress conditions (**Figure 8**).

Soluble sugar levels in different lines were quite similar under normal conditions. When exposed to salt and osmotic stress, the soluble sugar levels increased in all studied lines; however, the soluble sugar levels were increased highly in the OE lines compared with the WT and KO 4 and KO 5 plants, and both OE lines had the highest proline levels among all the lines. The KO 4 and KO 5 plants had the lowest proline levels compared with the OE and WT lines under salt and osmotic stress conditions (**Figure 8**).

## Discussion

AtRZFP is a RING/FYVE/PHD zinc finger-containing protein, which is suggested to have the function of zinc ion binding, according to the annotation in TAIR^2^. However, to date, the function of AtRZFP had received little attention. Additionally, the functions of AtRZFP or its homologs in response to abiotic stress were unknown. In the present study, we showed that AtRZFP regulates abiotic stress tolerance positively (**Figure 3**). However, AtRZFP plays a role in abiotic stress tolerance, and we found that a series of physiological responses related to abiotic stress tolerance (particularly to salt and osmotic stress) were changed when the expression of *AtRZFP* was altered.

Na^+^ is one of the most toxic ions, and high Na^+^ inhibits enzyme activity and disrupts K^+^ uptake, leading to K^+^ deficiency in the cytoplasm (Maathuis, 2009; Ma et al., 2011). Therefore, reduced Na^+^ accumulation is quite important for stress tolerance. In the present study, both CoroNa-Green staining and Na^+^ measurement showed that the expression level of *AtRZFP* is negatively correlated with Na^+^ accumulation (**Figure 6**), indicating that AtRZFP plays a role in preventing Na^+^ accumulation under salt stress conditions. Sufficient K^+^ is important for cellular osmotic adjustment and the activities of many enzymes (Zhu, 2003), and there is a strong positive correlation between salt tolerance and the ability to retain K^+^ in plants under salinity stress (Chen et al., 2005, 2007; Cuin et al., 2008). Our results showed that the transcription level of *AtRZFP* was positively correlated with K^+^ contents (**Figure 6**), suggesting that AtRZFP plays a role in preventing loss of K^+^ under salt stress. Therefore, expression of AtRZFP could reduce Na^+^ accumulation, and prevent K^+^ loss. The reduced Na^+^ accumulation might contribute to avoiding toxic ion damage. The relatively high level of K^+^ might play a role in maintaining cellular osmotic adjustment and enzyme activities, leading to significantly improved salt stress tolerance.

Stomatal regulation is a key process that links gas exchange and transpiration (Brodribb and Holbrook, 2003), and partial or complete closure of stomata could help plants to maintain a favorable water balance (Tombesi et al., 2015). Under abiotic stress conditions, reduction of the stomatal aperture is a common strategy employed by plants to avoid further water loss, and is important for plant survival (Moldau et al., 2011). Our study showed that overexpression of *AtRZFP* reduced the stomatal aperture, whereas knockout of *AtRZFP* increased the stomatal aperture (**Figures 7B,C**), suggesting that AtRZFP could affect the closure/opening of the stomatal aperture to maintain a favorable water balance, thereby enhancing salt and osmotic stress tolerance.

Some ZFPs mediate stress tolerance to adjust osmotic potential (Luo et al., 2012a; Wang et al., 2016). In the present study, the transcription of *AtRZFP* correlated positively with soluble sugars and proline levels, suggesting that AtRZFP might modulate the biosynthesis of soluble sugars and proline positively (**Figure 8**). Sugars and proline are both important compatible osmolytes in the adjustment of plants’ osmotic potential (Zhang X. et al., 2011); therefore, these results suggested that AtRZFP could adjust the osmotic pressure to control stress tolerance.

Some ZFP subfamilies play important roles in regulating ROS scavenging capabilities when exposed to abiotic stress (Davletova et al., 2005; Baek et al., 2015). Consistent with these studies, our results showed that the expression of *AtRZFP* decreased ROS accumulation and enhanced SOD and POD activities (**Figure 4**), suggesting that AtRZFP might improve ROS scavenging by increasing the activities of SOD and POD, leading to reduced ROS accumulation.

In the present study, to determine the function of AtRZFP, we used AtRZFP mutant line SALK_119330 for loss-of-function analysis, which is only one knock-down allele mutation plant. However, there was a shortcoming in study that we had not performed the complementary experiment. Namely, SALK_119330 plants were transformed with AtRZFP that is under the control of its native promoter. This complementary study will demonstrate that the observed phenotypes are indeed due to the mutation in AtRZFP, and need to be conducted in the future study.

## Conclusion

We showed that AtRZFP is involved in salt and osmotic stress tolerance. When exposed to abiotic stress, AtRZFP is induced by salt or osmotic stress. The expression of AtRZFP inhibited Na^+^ accumulation and reduced K^+^ loss. AtRZFP also controls the stomatal aperture to maintain the water loss balance. Additionally, similar to other studied ZFPs, RING/FYVE/PHD AtRZFP also enhances ROS scavenging and regulates the osmotic potential positively to confer abiotic stress tolerance.

## Author Contributions

YW designed research and wrote the paper. DZ performed research. DZ and HX analyzed data. WZ and HL contributed to genes expression analysis. YZ and XS cloned the gene. YW revised the manuscript. All the authors reviewed the manuscript. All authors have no competing financial interest.

## Conflict of Interest Statement

The authors declare that the research was conducted in the absence of any commercial or financial relationships that could be construed as a potential conflict of interest.

## Footnotes

1 http://rsbweb.nih.gov/ij

2 http://www.arabidopsis.org/
