# Supplementary material for: An Arabidopsis Zinc Finger Protein Increases Abiotic Stress Tolerance by Regulating Sodium and Potassium Homeostasis, Reactive Oxygen Species Scavenging and Osmotic Potential
Source: Front Plant Sci. 2016 Aug 24;7:1272. doi: 10.3389/fpls.2016.01272 (PMC4995212; doi:10.3389/fpls.2016.01272)
Supplement: Supplementary file 1 [file Table_1.DOC]

Supplementary Table 1: The primer sequences used in construction.

| **Gene** | **Forward primers (5'-3')** | **Reverse primers (5'-3')** |
| --- | --- | --- |
| pROK2-AtRZFP | CTCTAGAGGATCCCCATGGCTGATGATCAGAGTTCAAG | TCGAGCTCGGTACCCTCACTGCGGTGAAGCTTCAGGTTG |
| 35S-GFP-AtRZFP | ACGCGTCGACATGGCTGATGATCAGAGTTC | GGACTAGTCTGCGGTGAAGCTTCAGG |
