# Supplementary material for: An Arabidopsis Zinc Finger Protein Increases Abiotic Stress Tolerance by Regulating Sodium and Potassium Homeostasis, Reactive Oxygen Species Scavenging and Osmotic Potential
Source: Front Plant Sci. 2016 Aug 24;7:1272. doi: 10.3389/fpls.2016.01272 (PMC4995212; doi:10.3389/fpls.2016.01272)
Supplement: Supplementary file 2 [file Table_2.DOC]

Supplementary Table 2: The primer sequences used in real-time RT-PCR.

| **Gene** | GenBank Accession number or  Locus in TAIR | **Forward primers (5'-3')** | **Reverse primers (5'-3')** |
| --- | --- | --- | --- |
| *Act7* | AT5G09810 | CCAGCCATCGCTCATCGGAATG | CAGACACTGTATTTTCTCTCTG |
| *Tub2* | AT5G62690 | GCCAATCCGGTGCTGGTAACA | CATACCAGATCCAGTTCCTCCTCCC |
| *AtRZFP* | *AT5G62460* | CAATGGTGTTCACTTAGATTTG | CAGCTAGAGCAGCAGCTTCCTG |
| *SALK_119330* | *AT5G62460* | GGATCCAATCAATCTATT | CTTGTGGCATATTTCACAGG |
